# Draft Genome Sequence of Two *Sphingopyxis* sp. Strains, Dominant Members of the Bacterial Community Associated with a Drinking Water Distribution System Simulator

**DOI:** 10.1128/genomeA.00183-16

**Published:** 2016-03-31

**Authors:** Vicente Gomez-Alvarez, Stacy Pfaller, Randy P. Revetta

**Affiliations:** U.S. Environmental Protection Agency, Office of Research and Development, Cincinnati, Ohio, USA

## Abstract

We report the draft genomes of two *Sphingopyxis* sp. strains isolated from a chloraminated drinking water distribution system simulator. Both strains are ubiquitous residents and early colonizers of water distribution systems. Genomic annotation identified a class 1 integron (*intI1*) gene associated with sulfonamide (*sul1*) and puromycin (*pac*) antibiotic resistance genes.

## GENOME ANNOUNCEMENT

Members of the genus *Sphingopyxis* (*Alphaproteobacteria*) are strictly aerobic, chemoheterotrophic, yellow-pigmented, rod-shaped Gram-negative bacteria that contain glycosphingolipids as cell envelope components (1). *Sphingopyxis* spp. have been isolated from a variety of environments, including wetland (2), sludge (3), seawater (4), natural mineral water (5), and soil (6). Recent reports have confirmed the presence and dominance of *Sphingopyxis* spp. in drinking water distribution systems (DWDS) (7–9), and their ability as early colonizers to form biofilms (10, 11). Little information is available about their ecological role in DWDS or potential to cause public health problems (12, 13).

Two strains (H107 and H115) were isolated from a chloraminated DWDS simulator (11), by plating biofilm on R2A plates for 7 days at 27°C. Both strains were identified (100% sequence homology) as dominant members of the bulk water and biofilm community (7). Phylogenetic analysis of 16S rRNA genes indicated that strains H107 and H115 should be classified in the genus *Sphingopyxis* and were closely related to *Sphingopyxis soli* BL03 (99.5%) (14) and *Sphingopyxis chilensis* S37 (99.1%) (15), respectively.

Genomic DNA was extracted using the Ultra-Clean DNA microbial isolation kit (MoBio Laboratories, Solana Beach, CA) and sequenced by rapid mode sequencing on the HiSeq 2500 platform (Illumina Inc., San Diego, CA) using a paired-end 125 bp Nextera XT DNA library. Prior to assembly, libraries were (i) cleaned from contaminants (adapters, phiX, artifacts, and human), (ii) error corrected via Tadpole, (iii) normalized to ≤100×, (iv) removed of low (<6×) coverage reads, and (v) filtered to a minimum length read of 125 nucleotides (nt). Reads were processed using the software package BBMap v35.34 (http://sourceforge.net/projects/bbmap) and *de novo* assembly with SPAdes v3.5.0 (16). The draft genomes of strains H107 and H115 consist of 57 and 63 contigs for a total of 4,308,137 and 4,493,891 bp with a G+C content of 64.84 and 64.33%, respectively. Genome assemblies were annotated with Prokka v1.10 (17) available as an application in Illumina BaseSpace Labs. The genome sequence of strain H107 contains 4,244 genes, 4,191 coding sequences (CDSs), 3 rRNAs, and 50 tRNAs, and strain H115 contains 4,327 genes, 4,277 CDSs, 3 rRNAs, and 47 tRNAs.

The average nucleotide identity (ANI) between the two *Sphingopyxis* strains, a similarity index between two genomes (18), is 83.018% and was calculated using the online calculator available from EzGenome (http://www.ezbiocloud.net/ezgenome/ani). The proposed cut-off for species is 95% to 96% (19). Comparison against reference genomes estimated an ANI average of 82.931% with *Sphingopyxis alaskensis* RB2256 (20), 82.777% with *Sphingopyxis fribergensis* Kp5.2 (21), and 69.271% with *Sphingopyxis baekryungensis* DSM 16222 (22).

Genomic annotation of the environmental H115 strain confirmed the presence of the class 1 integrase (*intl1*) gene associated with the dihydropteroate synthase (*sul1*) gene, encoding resistance to sulfonamide (23). In addition, *sul1* gene was found linked to the puromycin-N-acetyltransferase (*pac*) gene. The *pac* gene encodes resistance to puromycin, an aminonucleoside antibiotic whose mode of action is distinct from the dihydropteroate synthase (24). Class 1 integrons are often embedded in plasmids and transposons, facilitating the lateral transfer of antibiotic resistance genes among bacteria (25).

### Nucleotide sequence accession numbers.

The whole-genome shotgun project has been deposited in DDBJ/ENA/GenBank under the accession numbers LNSJ00000000 and LNSA00000000. The versions described in this paper are the first versions, LNSJ01000000 and LNSA01000000.
